# Editorial: Genomic selection and characterization in cereals

**DOI:** 10.3389/fgene.2022.1092095

**Published:** 2023-01-11

**Authors:** Muhammad Abdul Rehman Rashid, Muhammad Sajjad, Alvina Gul, Yan Zhao

**Affiliations:** ^1^ Department of Agricultural Sciences, Government College University Faisalabad, Faisalabad, Pakistan; ^2^ Department of Bioinformatics and Biotechnology, Government College University Faisalabad, Faisalabad, Pakistan; ^3^ Department of Biosciences, COMSATS University Islamabad, Islamabad, Pakistan; ^4^ Plant Biotechnology Department, Atta-ur-Rahman School of Applied Biosciences (ASAB), National University of Sciences and Technology (NUST), Islamabad, Pakistan; ^5^ College of Agronomy, Shandong Agricultural University, Taian, Shandong, China

**Keywords:** genomic selection, cereals, characterization, rice, wheat, maize, sorghum, barley

## Introduction

Genomics is the branch of biological science in which the genomic content and structure of an individual genome are evaluated to ascertain expression and characterize its functions at the molecular, cellular, and cytological levels, and higher, in an ecosystem. Advancements based on multi-omic approaches have been reported in genetic and breeding studies of sustainable food security and climate-resilient crop improvements. The integration of biotechnology and bioinformatics has resulted in significant progress in identifying, analyzing, and verifying various structural, functional, and comparative genomic features in living beings.

Cereals have not only been selected as a staple food but also as a source of nutrients and income worldwide. The identification and verification of major and minor genes contributing to sustainable food security and resistance to biotic and abiotic stresses will be essential for breeding climate-resilient varieties and hybrids.

Peer-reviewed studies were collected on the latest trends, which covered three areas of genetics: forward, reverse, and comparative genetics. The articles characterized double haploids (DH), near-isogenic lines (NILs), diverse populations, segregating populations, and other domesticated germplasm, and presented models and marker-based genomic selection for improving crop yield and resistance to environmental stresses. These articles focused on rice, wheat, maize, and *Sorghum* crops.

## Wheat

Wheat is a major staple food grown in 89 countries and consumed by 2.5 billion people. Genetic improvement of wheat is indispensable for meeting the dietary requirements of a global population of ∼9.8 billion in 2050. The identification and induction of multi-ovary genes from mutant to semi-dwarf wheat lines through hybridization improved the grain yield potential of wheat Irshad et al. Three-pistil (multi-ovary) wheat is also a useful genetic resource for the commercial hybrid-seed production of wheat. Among insect pests, multivoltine insects, including saddle gall midge, orange, and yellow wheat blossom midges, fruit flies, and thrips are catastrophic for the environment in Europe. Arif et al. identified 246 QTLs resistant to these insects using genome-wide association studies (GWAS) in winter- and spring-wheat populations. The potential candidate genes were predicted to be involved in stomatal immunity and closure, and leaf-blade cuticular wax development, leading to the formation of physical barriers to insects. Some other candidates were predicted to be involved in the production and regulation of certain enzymes against stress stimuli. One study revealed two stable and five environment-specific QTLs for inducing stripe-rust resistance in wheat Tehseen et al.

Sandhu et al. explored the scope of multi-trait genomic selection (GS) models for predicting qualitative traits through cross-validation, independent-prediction, and independent-and-across-location prediction for a panel of 666 soft-white-wheat genotypes grown for 5 years. The results revealed that the overall prediction accuracies of the multi-trait GS model for within- and across-location prediction were 5.5% and 7.9% better, respectively, than the single or uni-trait models. Merrick et al. compared the regression and classification-based genomic selection models in winter wheat for the skewed phenotypes of infection type (IT) and stripe-rust-disease-severity (SEV). The best combination of relative efficiency and accuracy was found for the square root-transformed phenotypes using ridge-regression-best-linear-unbiased-prediction and support-vector-machine-regression models. The study concluded that breeders can accurately predict their breeding lines with skewed phenotypes by using non-parametric and linear regression models over combined years.

Tehseen et al. collected 600 bread-wheat landraces from eight ecological zones and characterized them with 11,830 high-quality SNPs Tehseen et al. The research suggested the model-based methods (DAPC and PCA) along with the STRUCTURE method is the best way for precise dissection of the population structure. The study explored the complex genetic architecture of studied landraces from the Fertile Crescent region using population structure analysis and estimation of genetic diversity.

Another study characterized 1,285 advanced breeding lines using historical multi-environment data for GS in breeding programs Ballén-Taborda et al. This study revealed that multi-institutional partnerships and genomics-enabled breeding are a useful approach for accelerating the varietal development process.

## Rice

Rice is the second top staple cereal food worldwide. The number of panicles per plant is a major yield component in rice. The nucleotide-binding adaptor shared by *APAF-1*, R proteins, and *CED-4 (NB-ARC)* plays a significant role in the structural development of plants, including panicle number. A study identified 258 members of the NB-ARC gene family in rice and characterized them for their structural, functional, and expression patterns. These genes were shown to be expressed in panicles, leaves, and roots, and regulated plant growth at panicle development stages. Among the NB-ARC genes, *GNP12* has been characterized as regulating rice yield by improving panicle features (panicle length and panicle grain number) and grain length in its eight major identified haplotypes Pan et al.

Another novel study evaluated multiple stress tolerance at the seed-germination stage to enhance the direct-seeded rice pattern. A total of 117 QTLs from 99 loci governing salinity, anaerobic, and combined (anaerobic and salinity) stress tolerance in rice were detected Islam et al. The study also observed two genes, *OsMT2B* (*Os01g0974200*) and *OsTPP7*, involved in multi-stress tolerance. Another study evaluated the genomic factors of low-temperature tolerance and the accumulation of essential minerals in a gene pool of near-isogenic lines (NILs) for genomic selection in japonica rice Ali et al. The study revealed the genomic regions associated with zinc, calcium, magnesium, chromium, phosphorous content, and low-temperature tolerance in rice at the booting stage Ali et al. The genomic regions significantly associated with chilling stress tolerance may not only help germplasm screening in rice breeding-targeted areas but also the biofortification of essential nutrients in grains. High cadmium accumulation in plants is a serious threat and known to cause cancer in humans. The GWAS-based characterization study of the rice genome found eight QTLs and 1,656 differentially expressed genes (DEGs), of which 799 and 857 DEGs were respectively expressed in root and shoot for Cd accumulation. A locus, *LOC_Os11g11050*, significantly associated with cadmium reduction was validated for marker-assisted genomic selection Wang et al.

Genomic selection by identification of the right parental combinations to maximize heterosis in hybrid breeding is also a well-known breeding and genetic tool. Hussain et al. revealed the temperature-sensitive genetic male sterility (TGMS) and cytoplasmic male sterility (CMS) lines and reported the best heterotic groups for hybrid rice breeding.

The reproductive stage in rice is vulnerable to drought causing a significant decrease in crop yield. Ahmad et al. characterized green super rice for morpho-physiological and molecular responses to drought at pre-anthesis. The germplasm was evaluated for certain drought-responsive genes (*OsDSM1*, *OsSADRI*, and *OsDT11*), and also mined for novel drought-responsive genes (*LOC_Os02g11960*, *LOC_Os11g36190*, *LOC_Os12g04500*, and *LOC_Os12g26290*) that enhance drought tolerance in rice breeding Ahmad et al.

Genomic selection by multi-trait (MT) genomic prediction is a useful tool for conserving phenotyping resources. It exploits the information from auxiliary or non-target traits and can enhance the prediction accuracy of target traits. Epistatic effects along with haplotype-based evaluation can improve the predictive ability in MT model genomic selection with additive effects Muvunyi et al.

## Maize

In maize, flowering time is among the most important agronomic traits that contribute to total yield. A study evaluated genomic variation and heterochromatic knob content Carvalho et al. and revealed that chromosome nine of the maize genome is associated with the heterochromatic knob that could reduce flowering time

## Barley

Barley (*Hordeum vulgare*) is the fourth most economically important cereal worldwide. Various genomic selection and characterization models have been studied in barley. However, the multi-parent advanced generation inter-cross (MAGIC) lines were found to be the most suitable for understanding the genetic basis of several traits and dissecting epistatic traits (Tao et al., 2022). This population, along with empirical analyses, was better than QTL mapping and/or epistatic effects at predicting grain yield.

## Sorghum

Two-component signal-transduction-system (TCS) genes assist plants in various physiological and cellular processes, such as cell division, leaf senescence, nutrition signaling, stress resistance, and chloroplast division. There are three types of proteins for developing these systems Irshad et al.: the response regulators (RRs) (Arif et al.), histidine kinases (HKs), and (Tehseen et al.) histidine phosphotransfer (HPs) proteins. A study on the *Sorghum bicolor* genome identified 37 TCS genes containing 13 HKs, five HP proteins, and 19 RRs (3 type-A, 7 type-B, 2 type-C, and 7 pseudo-RRs). Expression validation by qRT-PCR and RNA-seq confirmed the responsive expression of these TCS genes to salt and drought stresses in *Sorghum* leaves Zameer et al.
